# Artificial intelligence-based tools for patient support to enhance medication adherence: a focused review

**DOI:** 10.3389/fdgth.2025.1523070

**Published:** 2025-04-29

**Authors:** Zilma Silveira Nogueira Reis, Gláucia Miranda Varella Pereira, Cristiane dos Santos Dias, Eura Martins Lage, Isaias José Ramos de Oliveira, Adriana Silvina Pagano

**Affiliations:** ^1^ Health Informatics Center, Faculty of Medicine, Universidade Federal de Minas Gerais, Belo Horizonte, Brazil; ^2^ Department of Obstetrics and Gynecology, Faculty of Medical Sciences, Universidade Estadual de Campinas, Campinas, Brazil; ^3^ Department of Pediatrics, Faculty of Medicine, Universidade Federal de Minas Gerais, Belo Horizonte, Brazil; ^4^ Department of Gynecology and Obstetrics, Faculty of Medicine, Universidade Federal de Minas Gerais, Belo Horizonte, Brazil; ^5^ Arts Faculty, Universidade Federal de Minas Gerais, Belo Horizonte, Brazil

**Keywords:** prescriptions, machine learning, artificial intelligence, directive counseling, medication adherence

## Abstract

**Objective:**

Medication adherence involves patients correctly taking medications as prescribed. This review evaluates whether artificial intelligence (AI) based tools contribute to adherence-related insights or avoid medication intake errors.

**Methods:**

We assessed studies employing AI tools to directly benefit patient medication use, promoting adherence or avoiding self-administration error outcomes. The search strategy was conducted on six databases in August 2024. ROB2 and ROBINS1 assessed the risk of bias.

**Results:**

The review gathered seven eligible studies, including patients from three clinical trials and one prospective cohort. The overall risk of bias was moderate to high. Three reports drew on conceptual frameworks with simulated testing. The evidence identified was scarce considering measurable outcomes. However, based on randomized clinical trials, AI-based tools improved medication adherence ranging from 6.7% to 32.7% compared to any intervention controls and current practices, respectively. Digital intervention using video and voice interaction providing real-time monitoring pointed to AI's potential to alert to self-medication errors. Based on conceptual framework reports, we highlight the potential of cognitive behavioral approaches tailored to engage patients in their treatment.

**Conclusion:**

Even though the present evidence is weak, smart systems using AI tools are promising in helping patients use prescribed medications. The review offers insights for future research.

**Systematic review registration:**

https://www.crd.york.ac.uk/PROSPERO/view/CRD42024571504, identifier: CRD42024571504.

## 1 Introduction

The proper use of prescribed medications is crucial for disease management, affecting health outcomes and enhancing the efficiency of healthcare systems. The World Health Organization (WHO) defines adherence as the alignment of an individual's actions with healthcare professional recommendations (1). Nonadherence negatively impacts patient health and healthcare systems resulting in suboptimal outcomes, disease progression, unnecessary costs, and inappropriate treatment modifications. These consequences are particularly pronounced in resource-constrained settings, where nonadherence increases inefficiencies and contributes to higher morbidity and mortality (2).

Non-adherence to medication doses has been found in 50% of patients, with dramatic consequences on their management of chronic conditions (3). Poor medication adherence has also been reported in half of patients due to failure to take their medications as prescribed (4). This involves both underdosing and overdosing. Insufficient dosage can reduce therapeutic benefits, while overdosing—such as compensating for missed doses—may lead to adverse effects (5). Inconsistent adherence can result in a myriad of consequences, including increased adverse events, unnecessary hospitalizations, antimicrobial resistance, higher costs, disease progression, and treatment failure or death (4, 6). Medication errors remain a major concern for health systems because they threaten patient safety, particularly affecting people with low health literacy, the elderly, and those with mental health disorders, all of whom are especially vulnerable (7, 8).

There is increasing recognition that aspects beyond patients' control can affect medication adherence (9). Healthcare providers' communication and behavior significantly influence medication use, with common breakdowns including inadequate explanations of medication instructions, inadequate approaches to patients' reluctance to use medications and patients' beliefs regarding health and treatment (5). Given these issues, effective communication between prescribers and patients is critical. Adherence interventions should consider not only patients but also the broader healthcare context. Recent research highlights the importance of interventions that incorporate structural and counseling components and include appropriately qualified and motivated health professionals to promote medication adherence and persistence (5).

Electronic prescribing systems have largely gained preference in many countries, contributing to improve the accuracy and efficiency of medication management and eliminate problems with readability and access to prescriptions (10). While legibility, growing number of drugs available in the market, and drug interaction alerts are already targeted by digital technology, other challenges still demand smarter systems such as the need for personalized treatment and directions adapted to patients' literacy. To meet these challenges, electronic systems are integrated with other organizational tools, such as electronic health records and pharmacy information systems (11). The benefits include reduced healthcare costs, minimized prescribing errors, improved medication outcomes, increased patient safety, and enhanced clinical decision-making.

Recent advances in Artificial Intelligence (AI) have created new opportunities for addressing long-standing challenges in healthcare. AI refers to the use of computerized systems to model intelligent behavior with minimal human intervention (12). Retrospective studies using machine learning (ML) to predict medication adherence have gained ground (13, 14). However, AI algorithms in applications ranging from early detection and diagnosis to the management and treatment of medical conditions as well as the improvement of patient engagement still need to be tested (15). The integration of AI into prescribing systems offers significant opportunities as well as notable challenges, particularly in optimizing medication management, providing real-time prediction on patient adherence patterns based on clinical data, personalizing treatment regimens, and minimizing the risk of adverse drug interactions (16, 17).

The objective of this article was to review evidence on AI-based tools that contribute to adherence improvement, adherence measurement or avoidance of medication intake errors, helping patients take their medications safely and correctly, thereby improving medication adherence compared to standard practices.

## 2 Methods

We conducted a focused review following the Preferred Reporting Items for Systematic Review and Meta-Analyses (PRISMA) statement (18). The present review was registered on PROSPERO under the registration number CRD42024571504 (Available at: https://www.crd.york.ac.uk/prospero/display_record.php?ID=CRD42024571504). A research question was formulated and refined using the PI(E)COS framework: Can artificial intelligence approaches enhance prescription directions to help patients safely and correctly take medications, thereby improving medication adherence compared to standard practices?

### 2.1 Search strategy

The search strategy was formulated by a librarian and information science professional on July 20, 2024, in collaboration with the research group. The development of the strategy was unrestricted, provided the publication had an abstract in English. The databases searched included MEDLINE/Pubmed, Cochrane Library, Embase, SCOPUS, Web of Science, and Lilacs using descriptors and filters specific to each database. The following key terms and their variations were searched: (Patient OR Clients) AND (“Computer Reasoning” OR “Artificial Intelligence” OR “Machine Intelligence” OR “Computational Intelligence” OR “Transfer Learning” OR “Learning, Transfer” OR “Natural Language Processing” OR “Machine Learning”) AND (Prescription OR Prescriptions OR “Electronic Prescribing”) AND (“Medication Adherence” OR “Drug Adherence” OR “Medication Non-Adherence” OR “Drug Compliance”). The full search strategy is outlined in the Supplementary Information.

### 2.2 Eligibility criteria

The present review intends to summarize evidence regarding new generations of systems with AI-based adherence prediction models as long as they contribute to understanding or improving medication adherence outcomes or to mitigate outpatients' self-medication intake errors and improve medication adherence. Studies investigating patients prescribed medication by an authorized professional for self-administration; approaches based on AI tools and medication adherence or self-administration errors were included. In instances of duplicate publications or secondary analyses of included studies, the publication with the longest follow-up period or the most comprehensive information was selected. There were no restrictions on the types of study design eligible for inclusion.

The exclusion criteria were as follows: studies on AI tools targeting prevention of prescribers' errors such as alerts on drug interactions or polypharmacy, or support to prescribers in determining dosage, administration route, and frequency of use; studies focused solely on digital reminders to remind patients of their medication schedules without AI-based decision-making; and inaccessible full publication or without an abstract in English.

### 2.3 Outcomes

To address the research question, we define the outcomes as follows:

• The primary outcome is AI tool's advantages in improving medication adherence alone or compared to standard practices. The metrics to assess medication adherence were quantitative or qualitative, as reported in the primary studies.
• The secondary outcome is the comparison of medication self-administration errors between the intervention using AI tools and standard practices. The metrics to assess the outcome were the occurrence of self-medication errors using quantitative or qualitative results, as reported in the primary studies.

The medical term self-medication error refers to medication taken at the wrong time or dose, confused with other medications, or wrongly stored.

### 2.4 Selection of studies and extraction of variables

All articles retrieved from the electronic databases were organized using StaRt® (State of the Art through Systematic Review) software. After removing duplicated studies, the titles and abstracts were independently screened by two researchers (E.M.L. and C.S.D) according to the inclusion and exclusion criteria. References of review studies related to the topic were manually screened. The articles selected for full-text reading were subsequently independently evaluated by two researchers. A third researcher (Z.S.N.R) was considered in case of any disagreement between the researchers. When additional information was needed or questions arose about the data from selected articles, the authors were contacted. If no response was received by the submission deadline for the systematic review, the researchers reserved the right to exclude the paper and data.

Data from the studies included for quantitative and qualitative analyses were independently extracted by pairs of researchers (E.M.L. and C.S.D, Z.S.N.R and F.R.O) into the StaRt® software and organized into a database. The data were subsequently checked by a third researcher (Z.S.N.R). The extracted data referred to: the author and year of publication, country of origin of the study, main objectives, study design, population characteristics, type of systems used, metrics related to medication adherence, and indicators of self-medication errors, AI tools advantages. A complete list of the extracted variables is available in the Supplementary Information. All researchers underwent standardized training in screening and data extraction processes to ensure consistency in the review process. Given the heterogeneity in study designs and results, a meta-analysis was deemed infeasible. Therefore, the findings were summarized in a qualitative synthesis.

### 2.5 Evaluation of study quality

Two independent investigators (G.M.V.P. and F.R.O.) conducted the quality analysis of both randomized and non-randomized studies. Randomized studies were assessed using the Cochrane Handbook for Systematic Reviews of Interventions (19), classifying the risk of bias as “low”, “high”, or “some concerns” in the domains of bias arising from the randomization process; bias due to deviations from intended interventions; bias due to missing outcome data; bias in the measurement of the outcome; bias in the selection of the reported result. We used the Revised Cochrane Risk of Bias Tool (ROB2) (20). Non-randomized studies were evaluated using the Risk Of Bias In Non-randomized Studies of Interventions (ROBINS-I) (21), a tool that organizes and presents evidence related to bias across seven domains: confounding, selection of participants, classification of interventions, deviations from intended interventions, missing data, measurement of outcomes, and reporting of results.

Additionally, we applied the minimum report recommended by ESPACOMP Medication Adherence Reporting Guidelines framework (22), which provides structured recommendations for reporting adherence-related research, as Supplementary Table S4.

## 3 Results

### 3.1 Study selection

**Figure 1:**
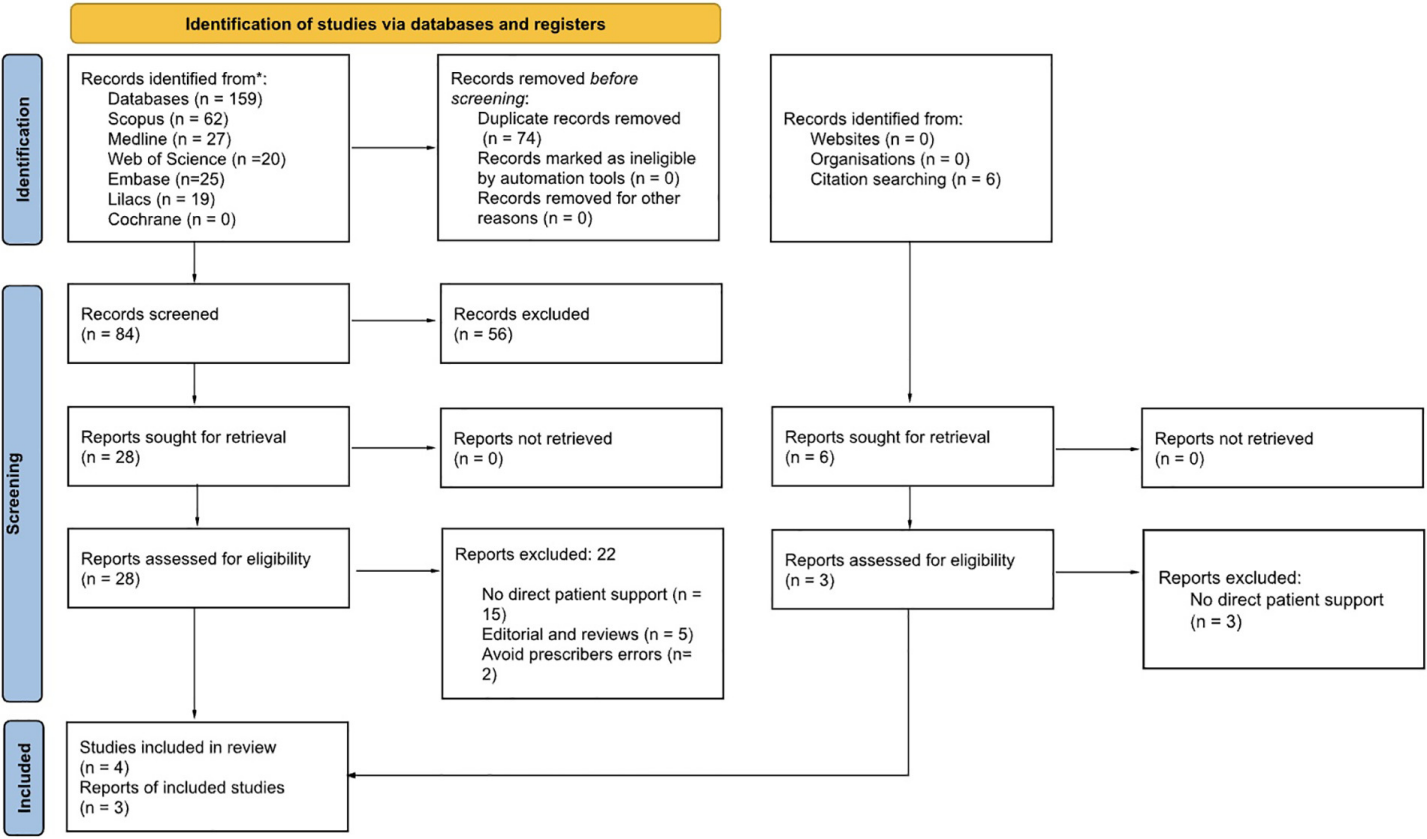
Flowchart of the review process. Adapted with permission from PRISMA 2020 flow diagram template for systematic reviews by Page et al., licensed under CC BY 4.0.

The search strategy retrieved 159 reports and six were identified by citation search. Upon cross-checking seven databases, 74 duplicates were removed (Figure 1). Only the Cochrane Library search yielded no results. Screening by title and abstract, all four reviewers selected 28 reports for complete reading. We identified six reports using a citation search; however, only three reports met eligibility criteria after full reading. During this step, we contacted the authors of two reports to clarify and ask questions regarding eligibility criteria and data extraction. We received a reply from only one of the reports' authors, and their clarification led to the exclusion of the report for not meeting the eligibility criteria. The second report was also excluded due to the lack of response from the author. At the end of the process, seven reports met the inclusion criteria. Studies without a focus on patient support to safely and correctly take medication were the main causes for exclusion. Detailed excluded studies, with reasons, are in Supplementary Tables S1, S2.

### 3.2 Study characteristics

Most studies included in this review were conducted in the United States, representing 5 out of 7 studies (71.5%), as shown in Table 1. Four studies involved real-world scenarios, including three clinical trials (23–25) and one prospective observational study (26). Collectively, these studies analyzed data from a total of 37,633 patients. Notably, the AiCure software platform was tested on 33,344 patients in a study by Gracey B, et al. (25), with an additional 75 patients in the study by Bain EE, et al. (23), both supporting private-sector healthcare applications. The majority of the selected studies focused on adult patients receiving medication for chronic disease management.

**Table 1:** Characteristics of included studies.

Authors, year	Country	Study design	Main objective	N	Target population	Disease or condition	Scenario used for experimentation	Comparisons
Bain EE et al., 2017 (23)	USA	Phase 2 multicenter, clinical trial, parallel-substudy.	To evaluate the use of a novel AI platform (AiCure) on mobile devices compared with mDOT for measuring medication adherence.	75 patients	Mean: 45.9 (10.86) years-old.	Schizophrenia, with a focus on cognitive impairment associated with the disease.	Private sector, multicenter.	Patient choice Intervention: 53 patients were monitored with the AI platform. Control: 22 patients using conventional mDOT.
Gracey, B et al., 2018 (25)	USA	Randomized, controlled, double-blind, clinical trial.	To evaluate whether AI-based medication adherence interventions, via live calls and tied to end-of-year Star Ratings and bonus payments, improve adherence rates compared to traditional targeting methods and no interventions (control).	33,344 patients (AI group)	Adults enrolled in Medicare.	Users of RASAs, OADs, and statins.	Private sector, regional.	Intervention: AI AllazoEngine identified which patients should receive live calls, mail, and faxes to the patient's prescribers (*n* = 33,344) Traditional Group: Patients who received live calls based on traditional methods (*n* = 5,423). Control Group: Patients without any intervention (*n* = 14,377).
Da Silva et al., 2019 (27)	Brazil	Conceptual framework tested in a simulated environment	To model, build and evaluate an intelligent system to assist patients with the ingestion of drugs and promote patient treatment adherence.	…	Elderly patients living alone or with a partner, in one residence connected to the Internet.	Hypertension with self-monitoring of blood pressure.	Tested by the research team themselves in a controlled laboratory setting (cabinet)	Proposed intervention: By notifying patients, relatives or caregivers, associating the blood pressure data with the history of medication intake, the system can indicate treatment adherence and help patients to achieve better treatment outcomes. No comparisons
Koesmahargyo et al., 2020 (26)	USA	Observational prospective study	The primary objective was to assess the accuracy of ML-based models in predicting medication adherence using real-time data from patients enrolled in clinical trials.	4,182 patients	Mean: 39.0 (11.7) years-old	A mix of primary conditions	Clinical research subjects and healthy population.	Exposition: AiCure software platform, computational vision using smartphones, following-up use, and correct doses. The study focused on refining predictive models, along the patients follow-up.
Julius, M.S et al., 2021 (29)	USA	Conceptual framework without real tests	To propose a framework to guide the design of ML-driven adherence and intervention systems to deliver intelligent intervention functions.	…	No real patients	The framework focuses on integrating non-clinical data (e.g., beliefs, attitudes, knowledge, and perception) from outpatients at their first clinic visit.	Theoretical model	Proposed intervention: reminder/alert/notification, information,education/training, motivation/reward, warning and risk analysis, knowledge, guidance and counseling, request and response tailored towards reducing patient's non-adherence to medication. No comparisons
Nayak, A. et al., 2023 (24)	USA	Randomized clinical trial	To examine whether a voice-based conversational AI application can help patients with type 2 diabetes titrate basal insulin at home to achieve rapid glycemic control.	32 patients	55.1 (12.7) (range, 30–74) years-old	Type 2 diabetes, English-speaking adults who required initiation or adjustment of once-daily basal insulin	4 primary care units, academic centers	Intervention: A custom voice AI software powered by Alexa®, that uses clinical protocols for insulin adjustments based on fasting blood glucose (FBG) levels. Control: Standard of care, with insulin titration management by the patient's clinician. Follow-up: 8 weeks.
Aparna, R. et al., 2023 (28)	India	Conceptual framework tested in a simulated environment	To develop a smart pill monitoring system that utilizes IoT sensors and ML models to enhance medication adherence by reminding patients to take their pills on time and notifying them when refills are necessary.	300 images per pill	Images of pills are divided into three different rotational angles of view.	Patients on long-term medication regimens.	Image studio and computational environment.	Proposed intervention: Enhance patient adherence using a stratified and proactive approach to different levels of non-adherence (high, moderate, slight, and non-adherence), enabling proactive interventions before non-adherence becomes critical. No comparisons

AI, artificial intelligence; ML, machine learning; mDOT, modified directly observed therapy; RASAs, renin-angiotensin system antagonists; OADS, oral anti-diabetics; USA, United States of America.

The clinical trials provided important comparisons between AI-based interventions and conventional treatment approaches (23, 24), while one of them, additionally, included a comparison with a group that did not receive any intervention (24).

In contrast, studies from Brazil (27), India (28) and one from the United States (29) were limited to simulation-based approaches, without testing in real-world settings.

### 3.3 Results of syntheses

Studies reporting results of AI tools assisting patients in correctly taking their medication made use of various technologies, as summarized in Table 2. Characteristics of AI-based tools are detailed in Supplementary Table S3. These included mobile applications (23, 26), smart call centers with live call interventions (25), and conversational AI platforms like Amazon Alexa® (24).

**Table 2:** Characteristics of solutions using AI tools and outcomes.

Authors, year	Digital health approach	Solution using AI tools	Adherence parameters	Adherence outcomes	Self-medication errors	AI advantages
Bain EE et al., 2017 (23)	The AI mobile app (AiCure)	AI Platform on Mobile Devices containing: Facial recognition: Used to identify a patient's identification. Confirms medication ingestion. Automated real-time data transmission: Adherence data, including ingestion confirmation, is sent to cloud-based dashboards for real-time monitoring and intervention. Alerts: Used for suspicious behavior detection as the drug or incorrect usage. Cumulative adherence tracking: allows for cumulative adherence tracking, helping researchers assess long-term adherence trends over the course of the study.	(1) visual confirmation of ingestion using the AI platform app, (2) self-reported dose via the self-report button in the app (no visual confirmation), (3) Self-reported dose over the phone to the study coordinator, (4) missed dose, (5) skipped dose, (6) dose taken in clinic. (7) Pharmacokinetic adherence through ABT-126 blood concentration measurements.	Intervention group: Cumulative pharmacokinetic adherence over 24 weeks was 89.7% (SD 24.92). Control group (mDOT): Cumulative pharmacokinetic adherence over 24 weeks was 71.9% (SD 39.81).	Suspicious drug administration behavior flagged by the AI platform: 19 (35.8%) subjects in the intervention group.	(1) Improvement in the patient's adherence rates compared to traditional methods like mDOT. (2) Real-time patient monitoring. (3) Opportune detection of suspicious behaviors (4) Potential to scalability
Gracey, B et al., 2018 (25)	Call center/AllazoEngine (proprietary)	AI interventions gathering: Predictive analytics: Identified patients at risk of non-adherence and those likely to benefit from interventions. Live call interventions: Calls were delivered to patients based on AI recommendations. Evolution: System continuously learns, improving the engine's accuracy.	Calculation based on pharmacy claims by the percentage of days a patient had medication available over a year. Patients with a PDC greater than 80% in a year-end were classified as adherent. Targets: (1) The patient's probability of being qualified for the Star Ratings medication adherence metrics at year-end. (2) The patient's risk of becoming non-adherent. (3) The patient's probability of positively changing his or her adherence given a live call intervention.	Intervention vs. Control group^a^: The AI group was 6.1% more likely to be adherent than the control group (*p* = 0.04). Intervention vs. Traditional group: The AI group was 7.8% more likely than the traditionally targeted group (*p* = 0.08). Control group vs. Traditional group: There was no significant difference in adherence between groups (*p* = 0.73).	No	(1) Improvement in the patient's adherence rates compared to traditional methods and any-intervention. (2) Cost-effectiveness: Better adherence outcomes with less intervention. (3) Predictive accuracy enhancing: The AI engine improved in accuracy throughout the study (from 86.9% in May to 97.7% by October 2016). (4) Scalability: AI systems like the AllazoEngine offer significant scalability advantages.
Da Silva et al., 2019 (27)	Not implemented. The system demands smart home TV systems integrated with IoT devices.	Intelligent system containing: Patient monitoring: Integrated sensors to smart devices allow checking medication adherence. Reasoning layer: The AI solution uses decision tree algorithms (J48, RepTree, RandomTree) to classify whether patients are adhering to their prescribed medication schedules. Messaging & Notifications: Patients receive reminders through smart TVs and smartphones, ensuring they follow their treatment plan. The interactive system automatically interacts with physicians, caregivers and families. Commercial Devices & IoT: It uses off-the-shelf devices and IoT infrastructure for a cost-effective and accessible solution in home environments.	(1) Timeliness, by checking if the patient took their medication at the prescribed time. (2) Accuracy of medication taken, based on correct medication and correct dosage. (3) Tracking of missed doses (4) Blood Pressure Monitoring: self-monitoring of blood pressure was part of the adherence metrics.	The adherence itself was not directly measured in real patients.	The experimental smart cabinet verified which medication was removed and whether it matched the prescription, reducing the risk of errors. AI algorithms process data to classify adherence and optimize treatment outcomes.	Potential advantages: 1) IoT integration with home devices makes the system user-friendly for elderly patients. (2) Opportune detection of correct medication at the right time, potentially reducing errors. (3) Personalized device for sending reminders, improving patient engagement with the treatment. (4) Scalable, flexible, cost-effective, and intelligent patient-centric intervention.
Koesmahargyo et al., 2020 (26)	The AI mobile app (AiCure)	ML algorithms providing: Patient monitoring: The AiCure platform as a smartphone-based application employs computer vision and ML to monitor and predict medication adherence through video recording of patients during dosing events. Predictive analytics: The core AI element is the XGBoost classifier, which is based on decision-tree algorithms and predicts adherence using real-time and historical adherence data. The model leverages features like condition, interventions, dose timing, and trial length.	Binary indicator of whether or not a patient took all prescribed medications during a given dosing event. (1) Daily Adherence Calculation: Percentage of doses taken as prescribed over a given time period. Non-adherence was flagged using alerts, such as “red alerts” for strong evidence of non-adherence and “orange alerts” for suspicious behavior. (2) Adjusted Adherence: Final adherence rate was adjusted based on the AI system's findings and human review of flagged events, providing an accurate daily adherence value. (3) Human Review: In cases where the AI flagged suspicious behavior (e.g., not taking the medication properly), human reviewers would check the videos to verify whether the medication was truly ingested or whether there was non-adherence or deceptive behavior. (4) Interventions and Patient Engagement: Study coordinators could intervene when non-adherence was detected, using methods such as texts, phone calls, or in-person visits. These interventions address adherence issues and contribute to valuable data, enhancing the predictive models.	Adherence Accuracy of classification models accurately predicted adherence across the trial (AUC = 0.83), the subsequent week (AUC = 0.87) and the subsequent day (AUC = 0.87).	Indirectly support the prevention of errors due to missed or improper doses by closely monitoring medication adherence and flagging non-compliance or suspicious behavior.	(1) Improvement in the patient's adherence rates, the prediction of overtime during the follow-up of patients (2) Real-time patient monitoring. (3) Opportune detection of suspicious behaviors (4) Resource optimization: Allows healthcare providers to allocate resources more efficiently by focusing on high-risk patients, reducing unnecessary interventions for those with consistent adherence.
Julius, M.S et al., 2021 (29)	Not Implemented (ongoing project)	ML framework gathering: ANF Inference System: To patient-specific predictions based on non-clinical data. Personalized intervention systems: Aimed to deliver health belief theory-inspired functions tailored to individual patient needs.	Conceptual framework to assess four levels of adherence: high, moderate, slight, and non-adherence.	The adherence itself was not directly measured in real patients.	Indirectly reduce errors by delivering tailored interventions based on predicted risk levels.	Potential advantages: (1) Personalized prediction and classification of non-adherence using patient beliefs and attitudes. (2) Opportune real-time intervention delivery, such as SMS, voice calls, and mobile applications. (3) Stratified and proactive approach.
Nayak, A. et AL., 2023 (24)	LLM voice AI generator	Voice-based conversational AI application containing: Voice-based interactions: Custom voice AI software - HIPAA-compliant conversational AI platform by Amazon, interacts with patients for reporting FBG levels. Automated insulin dose adjustments based on clinical algorithms approved by clinicians. Real-time data tracking is available to clinicians via a web portal. Emergency protocols for managing hypoglycemia and hyperglycemia.	(1) Insulin adherence calculated based on logged data from participants. The final adherence was the percentage of days the patients correctly followed their insulin regimen over the 8-week trial period.	Insulin adherence, mean (SD), 82.9% (20.6) using IA-voice vs. 50.2% (43.0) standard of care. Difference 32.7% (8.0–57.4), *p* = 0.01 Without impact in attitudes toward medication adherence score, mean 0.8 (4.3) using IA-voice −0.1 (2.6) standard of care. Difference 1.0 (−1.6–3.5), *p* = 0.46	Indirectly, minimizing self-medication errors by providing daily insulin adjustments, offering real-time guidance, and incorporating safety protocols to manage hypoglycemia and hyperglycemia	(1) Improvement in the patient's adherence rates compared to traditional methods. (2) Preventive approach: opportune detection of suspicious behaviors. (3) Reduction in diabetes-related emotional distress compared to the control group. (4) Real-time data tracking. (5) Reduction in clinical visits.
Aparna, R. et al., 2023 (28)	Not Implemented Demands smart pill bottle, sensors and camera	IoT-based system leverages wireless networks, cloud computing, and ML gathering: Patient monitoring: Smart pill bottle, weight sensors, and a camera module to track pill consumption, pill count, and pill recognition. YOLOv4 ML pre-trained model used for pill detection and recognition. Weight sensors to track the number of pills left in the bottle. IoT architecture (using ESP32 microcontroller and cloud-based data processing) for real-time notifications and data transfer to a cloud platform. Real-time alerts are sent via notifications using IFTTT and email if the patient misses a dose or if the number of pills drops below a predefined threshold.	The system design allows to measure adherence using a combination of IoT sensors and ML: (1) Timely consumption of medication (tracked by the opening of the pillbox and removal of pills). (2) Accuracy of medication (verified through pill recognition). (3) Real-time monitoring to ensure that doses are not missed, along with alerts and reminders (such as refill reminders) to support adherence.	The adherence itself was not directly measured in real patients. Expected results: Enhance patient adherence since the system's performance in detecting and counting pills achieved 97% classification accuracy for pill recognition, with a margin of error of ±1 pill for pill counting.	Potential since the system was designed to help patients avoid self-medication errors.	Potential advantages: (1) Improvement in the patient's adherence with high accuracy to ensure patients take the correct medication. (2) Timely real-time intervention provided by IoT sensor data. (3) Cloud-based scalability by using cloud computing to store and analyze data of larger patient populations, which can support broader healthcare applications and insights.

ANF, adaptive neuro-fuzzy; AUC, area under curve; FBG, fast blood glucose; HIPAA, health insurance portability and accountability act; IFTTT, IF this then that; ML, machine learning; PDC, proportion of days covered; TV, televisor; Vs., vs.; YOLOv4, you only look once at version 4.

^a^ Only 3,903 patients (11.7%) of the patients successfully received AI interventions.

Studies exploring solutions in simulated scenarios made use of an intelligent system with wireless integration with home devices systems (27), a user-friendly IoT-based system with cloud computing, ML wireless integration systems with smart pill bottles, sensors, and cameras (28), and predictive ML framework of models to trigger real-time actions alerted by intervention, such as SMS, voice calls, and interaction by mobile applications (29).

A common requirement across the selected studies was targeting the need for timely interventions. To achieve this, data input often began with real-time patient monitoring, utilizing technologies like an AI Platform accessible on mobile devices for patient identification and medication ingestion confirmation using ML algorithms (23, 26, 27). In other cases, voice interaction played a crucial role, particularly in voice-based conversational AI application (24), and interventions using AI in AllazoEngine-powered call center (25). These systems often integrated alerts that provided feedback to patients, caregivers, doctors, or call centers, particularly in cases of suspicious behavior, such as incorrect medication use.

Framework-based reports explored further possibilities of intelligent systems for patient monitoring using IoT-integrated sensors and smart devices to track medication adherence (27). Systems incorporating smart pill bottles, weight sensors, and camera modules for tracking pill consumption, pill count, and pill recognition were found to be potentially valuable for generating data. These data are meant to be prospectively used by ML algorithms, such as the YOLOv4 ML pre-trained model, for pill detection and recognition (28).

Clinical trial outcomes demonstrated a promising impact of systems using AI tools on patient medication adherence, with different studies employing various metrics to measure success. In the randomized clinical trial by Gracey B. et al. (25), the AI group was found to be 6.1% more likely to adhere to their treatment compared to the control group (*p* = 0.04). In a prospective cohort study, Koesmahargyo et al. (26) reported that the accuracy of the XGBoost classifier algorithm in predicting patient adherence improved over time, utilizing both real-time and historical adherence data based on AI Cure mobile application. Using the same proprietary mobile application, Bain EE et al. reported cumulative pharmacokinetic adherence over 24 weeks of 89.7% (SD 24.92) in the group using AI Platform on mobile device interventions, compared to 71.9% (SD 39.81) in the control group (23). Although this study did not provide formal statistical comparisons, both systems demonstrated the ability to deliver real-time patient monitoring, enabling timely detection of medication misuse. Another clinical trial, using a voice-based conversational AI application to support type 2 diabetes patients, conducted by Nayak A. et al. (24), showed that insulin adherence rates were 32.7% higher in the AI-voice application compared to the standard care group (95% Confidence Interval from 8.0% to 57.4%, *p* = 0.01). Furthermore, we extracted information from selected studies that reveal the potential scalability of AI tool solutions identified in this review, particularly in reducing the need for clinical visits and enabling healthcare providers to focus on high-risk patients prone to non-adherence. Table 2 presents the advantages of AI tools from our perspective analysis.

Studies without real-world care scenario testing proposed intelligent systems using ML algorithms and wireless networks with the potential to assist patients in avoiding self-medication errors (27, 28). One study focused on processing data to classify adherence and optimize treatment outcomes (29).

### 3.4 Risk of bias in studies

**Figure 2:**
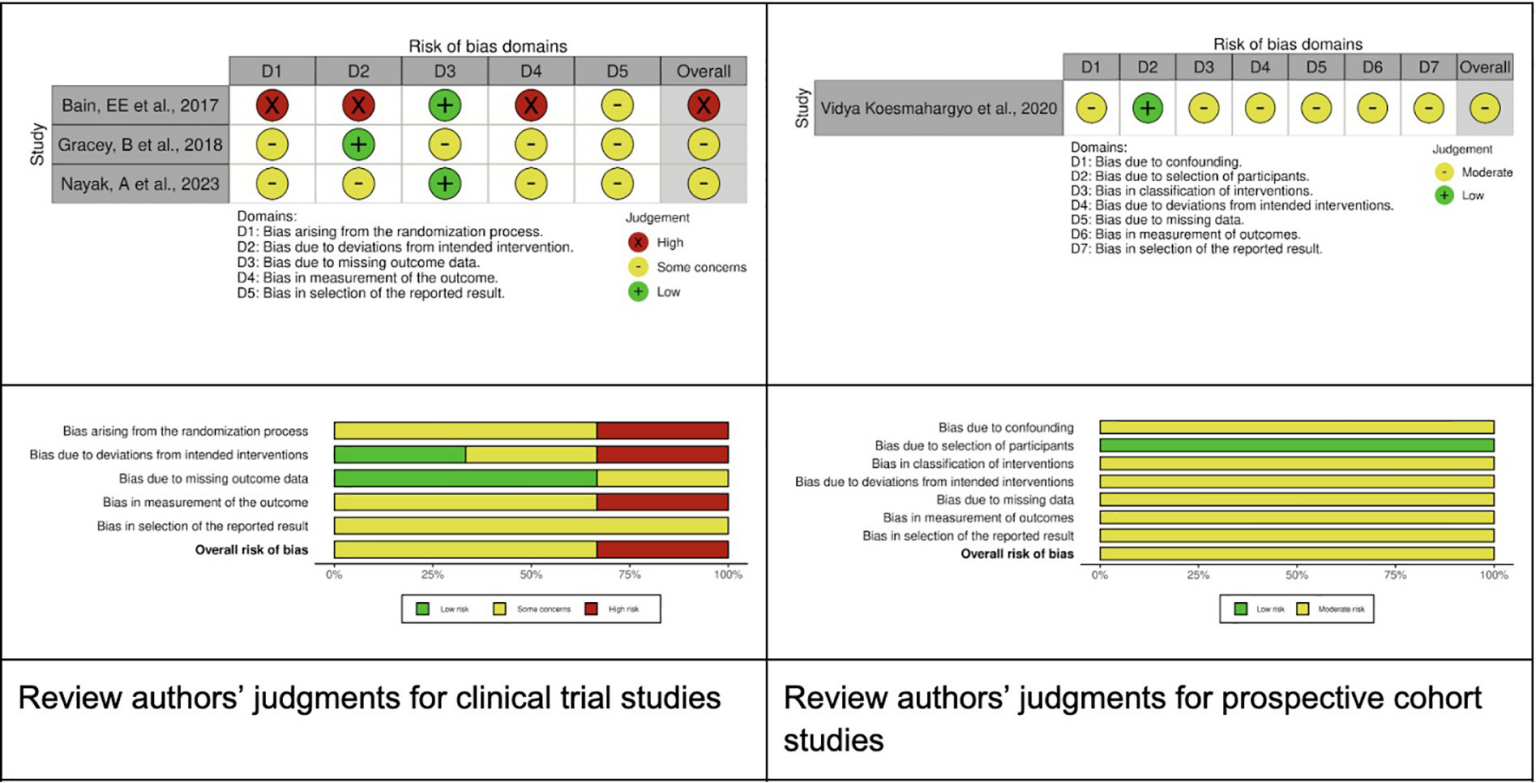
Risk of bias summary.

Figure 2 illustrates the evaluation of bias risk utilizing the Revised Cochrane Risk of Bias Tool (ROB2) (20), and the Risk of Bias in Non-randomized Studies of Interventions (ROBINS-1) (21), as delineated in the Cochrane Handbook for Systematic Reviews of Interventions (19).

The randomization process presented some concerns in two studies and was classified as high-risk in one. Deviation from the intended intervention was assessed as high risk in one study, low risk in another, and raised some concerns in a third study. The risk associated with missing outcome data was deemed low in two studies and presented some concerns in one study. The measurement of outcomes was classified as high risk in one study (24) and moderate in two others (23, 25). Lastly, the selection of reported results was evaluated as moderate across all three studies. Considering the ROBINS-I assessment, we classified one study (26) as an overall moderate risk of bias.

Adherence measurement quality according to the Emerge checklist (22) was presented in Supplementary Table S4.

## 4 Discussion

### 4.1 Main findings

Evidence regarding AI tool interventions directly benefiting patient medication adherence or reducing self-medication errors is still limited. The lack of measurable outcomes based on well-controlled studies and moderate to low quality of evidence emerged as a relevant issue in this focused review. We may account for this scarcity of studies due to the relatively recent introduction of AI tools in real healthcare settings (30) and the inherent complexity of implementing innovations that influence patient behavior (31). However, our protocol's comprehensive search strategy, which included six databases without restrictions on language or publication year, provided that the title and abstract were in English, ensured a broad and inclusive scope. This way, we alert the need for further studies demonstrating an effective use of AI tools for this proposal.

To discuss our results, we considered real-world scenario studies and studies based on simulations separately. Regarding real-world scenario studies, all four studies conducted in real healthcare environments were within the private sector in the USA, using prospective designs. Digital companies have historically led the AI tools introduction in healthcare (15, 32). Despite a large investment from technology companies, experts, and researchers, the true impact of AI algorithms on improving people's health remains difficult to measure. One of the challenges in evaluating smart systems performance is designing studies that ethically determine the usefulness of intervention with real-time judgments, preferably using a blinded and randomized approach compared with current practices (15).

The randomized clinical trials, which we classified as moderate risk of bias, compared groups of standard care, and control without interventions with users of smart systems using AI tools. These trials demonstrated the potential of ML models to timely identify patients at high risk of non-adherence and deliver opportune alert interventions through interactions via call centers (25) or custom voice AI generator (24). It is important to recognize that AI tools were not the sole driver of positive outcomes in these trials; rather, it was a component of broader patient surveillance strategies. For instance, in Gracey B, et al. report, although the difference between the AI tools intervention was 6.1% more likely to be adherent compared to the control group without any intervention (*p* = 0.04), the AI tools intervention had no significant effect when compared to traditional live-call monitoring (*p* = 0.08) (25). This implies that while AI can enhance adherence, its impact may be modest and often works in conjunction with other aspects of patient care such as surveillance using call centers itself.

The outcomes from Bain EE, et al. (23) had limited value since it included only a few patients and no comparative statistical test between the AI Platform on mobile devices intervention and the control group. Furthermore, the distribution of patients between intervention and control groups was not randomized, leading to a classification as high risk of bias. Despite this, the study reported a value of 35.8% detection of suspicious incorrect drug administration behavior flagged by the AI platform, in the intervention group. Even so, progress is expected in intelligent systems with AI in this area of healthcare, as they can improve accuracy over time, as presented by Koesmahargyo et al. (26) real-time ML algorithms predicting adherence among a mix of primary conditions. As a limitation, we noticed such progress had no confidence intervals or statistical comparisons for the model's accuracy by Area Under Curve (AUC), and the risk of bias risk was considered moderate.

Regarding bias risk in studies based on conceptual frameworks, the quality of evidence was not evaluated due to the absence of real scenario outcomes and intervention vs. control comparisons. It should be noted that following our review protocol (PROSPERO 2024 CRD42024571504), which included overall study designs, we decided to include studies on conceptual frameworks, as long as they met our inclusion criteria. Even though these studies are based on simulation, their outcomes were useful in pointing to the relevance of personalizing the interaction with each patient, considering patient beliefs and attitudes (29), using remote, real-time measurement of medication intake with IoT-based systems and ML algorithms, and allowing for proactive clinical intervention to optimize health outcomes (27, 28). The AI synergy with the Internet of Things (IoT) promises to enhance the next generation of smart systems in healthcare providing real-time surveillance (33).

Studies involving retrospective analysis of databases to predict medication adherence were excluded (Supplementary Tables S1, S2). Despite their potential for further providing smart interventions, our interest was to look for AI systems that can interact in a personalized way with patients, helping them with drug treatment and measuring adherence or avoiding self-administration errors. In this review, we opted for a broad search strategy combined with well-defined eligibility criteria to ensure the inclusion of a diverse range of AI-based interventions, expecting that AI-based approaches would leverage features that characterize patients, their conditions, and their relationship with prescribed medications providing solutions to the targeted strategies.

### 4.2 Limitations of evidence

We encountered different methods for measuring medication adherence, which was a challenge to synthesize the data extracted in this review and to interpret the results. While only seven studies met the inclusion criteria, this reflects the current state of evidence. The heterogeneity in the included studies evolving different applications based on AI tools reinforces the need for more standardized and well-controlled research. Although there is a report of a psychometric scale to measure treatment adherence, this has not been used since 2019 and was not used in any of the selected studies (34). The expected synthesis using the meta-analysis included in our review protocol was not possible. Medication nonadherence is a multifactorial issue influenced by patients' ability to remember their medications, the clarity of instructions, and the complexity of treatment regimens (5). These factors often contribute to unintentional nonadherence, also referred to as passive nonadherence, which stems from forgetfulness or carelessness. This relevant nonadherence perspective was not a concern in most of the selected studies.

Additionally, patients' beliefs about medications, and their perceived view of medication necessity and affordability, also play a role (35). Moreover, intentional nonadherence is increasingly acknowledged as a significant factor, with its determinants being complex and including cost, adverse effects, patient preferences, disagreement on treatment necessity, and communication breakdowns between patients and providers (36). In this review, the study by Julius et al. extended the analysis of adherence beyond the simple act of taking or not taking medication, exploring the integration of non-clinical data, such as patients' beliefs, attitudes, knowledge, and perceptions, into a conceptual model (29). Their study highlighted the potential of smart systems to personalize interventions, delivering functions inspired by health belief theory to address individual patient needs.

Another important issue raised in our review was the diversity of clinical conditions covered by the studies, limiting generalization based on outcomes. It is essential to recognize that different disease groups present unique challenges to achieving patient adherence. For example, systems designed to support patients with schizophrenia (23) focused on addressing cognitive impairment associated with the condition. In contrast, the voice-based conversational AI application developed by Nayak et al. was tailored to help patients with type 2 diabetes titrate basal insulin at home, aiming to achieve rapid glycemic control (24). Furthermore, it is important to consider that the number of patients involved in the studies varied from 32 to 33,344 exposed to AI-based tools.

Based on the EMERGE framework (22), we found substantial variability in how adherence was defined, and measured. We reported across studies, which has important implications for the interpretation of our review findings (Supplementary Table S4). Studies in the phase of implementation directly measured adherence behavior through pharmacy claims data (25), AI-based video monitoring (23, 26), and self-reported adherence logs (23). In contrast, others relied on predictive models to provide early intervention for high-risk patients of non-adherence (29) or IoT-based adherence tracking still in prototyping (27, 28), which were not tested in real-world patient populations. These inconsistencies were limiting factors to allow comparability between studies and introduced heterogeneity into our findings. At the same time, this analysis was important to recommend that future studies ensure more transparency in the way they report medication adherence.

## 5 Conclusion

Based on this review, the evidence supporting AI tools to assist patients in adhering to prescribed medications is still weak. Part of the outcomes are influenced not solely by systems using AI-based tools but also by their integration into smart elements within already effective monitoring practices. Nonetheless, this review highlights AI's growing influence in healthcare and offers insights for future research. It underscores the current applications of AI in medication adherence while identifying key areas for further exploration and development.

## Data Availability

The datasets presented in this study can be found in online repositories. The names of the repository/repositories and accession number(s) can be found in the article/Supplementary Material.
