# Supplementary material for: Artificial intelligence-based tools for patient support to enhance medication adherence: a focused review
Source: Front Digit Health. 2025 Apr 29;7:1523070. doi: 10.3389/fdgth.2025.1523070 (PMC12069381; doi:10.3389/fdgth.2025.1523070)
Supplement: Supplementary file 1 [file Datasheet1.pdf]

Supplementary Material

Artificial Intelligence for Patient Support to Enhance Medication Adherence: A Focused Systematic Review

Zilma Silveira Nogueira Reis[1]*, Gláucia Miranda Varella Pereira[2], Cristiane dos Santos Dias[3], Eura Martins Lage[4], Isaias José Ramos de Oliveira[1], Adriana Silvina Pagano[5]

[1]Health Informatics Center, Faculty of Medicine, Universidade Federal de Minas Gerais, Belo Horizonte, Brazil

[2]Department of Obstetrics and Gynecology, Faculty of Medical Sciences, Universidade Estadual de Campinas, Brazil

[3]Department of Pediatrics, Faculty of Medicine, Universidade Federal de Minas Gerais, Brazil

[4]Department of Obstetrics and Gynecology, Faculty of Medicine, Universidade Federal de Minas Gerais, Belo Horizonte, Brazil

[5]Arts Faculty, Universidade Federal de Minas Gerais, Belo Horizonte, Brazil.

*** Correspondence:** Zilma Reis: zilma@ufmg.br

Supplementary Information - Search Strategy

Search date: 07/20/2024

**MEDLINE/PubMed (27 articles)**
(Patient OR Clients OR Client OR "Clinic Visits" OR "Clinic Visit") AND ("Intelligence, Artificial" OR "Computer Reasoning" OR "Reasoning, Computer" OR "Artificial Intelligence" OR "Machine Intelligence" OR "Intelligence, Machine" OR "Computational Intelligence" OR "Intelligence, Computational" OR "Learning, Machine" OR "Transfer Learning" OR "Learning, Transfer" OR "Language Processing, Natural" OR "Language Processings, Natural" OR "Natural Language Processings" OR "Processing, Natural Language" OR "Processings, Natural Language" OR "Machine Learning") AND ("Adherence, Medication" OR "Drug Adherence" OR "Adherence, Drug" OR "Medication Persistence" OR "Persistence, Medication" OR "Medication Nonadherence" OR "Nonadherence, Medication" OR "Medication Non-Adherence" OR "Medication Non Adherence" OR "Non-Adherence, Medication" OR "Medication Noncompliance" OR "Noncompliance, Medication" OR "Medication Compliance" OR "Compliance, Medication" OR "Drug Compliance" OR "Compliance, Drug" OR "Medication Non-Compliance" OR "Medication Non Compliance" OR "Non-Compliance, Medication" OR "Administration, Self" OR "Administrations, Self" OR "Self Administrations" OR "Therapeutic Adherence and Compliance" OR "Treatment Adherence" OR "Adherence, Treatment" OR "Therapeutic Adherence" OR "Adherence, Therapeutic" OR "Medication Adherence" OR "adherence monitoring" OR "adherence prediction") AND (Prescription OR Prescriptions OR "Electronic Prescribing"OR "Prescribing, Electronic")

COCHRANE LIBRARY (0)

(Patient OR Clients OR Client OR "Clinic Visits" OR "Clinic Visit") AND ("Intelligence, Artificial" OR "Computer Reasoning" OR "Reasoning, Computer" OR "Artificial Intelligence" OR "Machine Intelligence" OR "Intelligence, Machine" OR "Computational Intelligence" OR "Intelligence, Computational" OR "Learning, Machine" OR "Transfer Learning" OR "Learning, Transfer" OR "Language Processing, Natural" OR "Language Processings, Natural" OR "Natural Language Processings" OR "Processing, Natural Language" OR "Processings, Natural Language" OR "Machine Learning") AND ("Adherence, Medication" OR "Drug Adherence" OR "Adherence, Drug" OR "Medication Persistence" OR "Persistence, Medication" OR "Medication Nonadherence" OR "Nonadherence, Medication" OR "Medication Non-Adherence" OR "Medication Non Adherence" OR "Non-Adherence, Medication" OR "Medication Noncompliance" OR "Noncompliance, Medication" OR "Medication Compliance" OR "Compliance, Medication" OR "Drug Compliance" OR "Compliance, Drug" OR "Medication Non-Compliance" OR "Medication Non Compliance" OR "Non-Compliance, Medication" OR "Administration, Self" OR "Administrations, Self" OR "Self Administrations" OR "Therapeutic Adherence and Compliance" OR "Treatment Adherence" OR "Adherence, Treatment" OR "Therapeutic Adherence" OR "Adherence, Therapeutic" OR "Medication Adherence" OR "adherence monitoring" OR "adherence prediction")
AND (Prescription OR Prescriptions OR "Electronic Prescribing"OR "Prescribing, Electronic")

Embase (25 articles)

(Patient OR Clients OR "patient participation" OR "Clinic Visits" OR "Clinic Visit") AND ("automated reasoning" OR "Artificial Intelligence" OR "Computational Intelligence" OR "machine learning" OR "Transfer of Learning" OR "Processing, Natural Language" OR "Machine Learning") AND ("Adherence, Medication" OR "medication compliance" OR "Medication Persistence" OR "Medication Nonadherence" OR "Compliance, Medication" OR "Drug Compliance" OR "Administration, Self" OR "Self Administrations" OR "Therapeutic Adherence and Compliance" OR "Treatment Adherence" OR "Adherence, Treatment" OR "Therapeutic Adherence" OR "Adherence, Therapeutic" OR "Medication Adherence") AND (Prescription OR Prescriptions)

SCOPUS (62 articles)
(Patient OR Clients OR Client OR "Clinic Visits" OR "Clinic Visit") AND ("Intelligence, Artificial" OR "Computer Reasoning" OR "Reasoning, Computer" OR "Artificial Intelligence" OR "Machine Intelligence" OR "Intelligence, Machine" OR "Computational Intelligence" OR "Intelligence, Computational" OR "Learning, Machine" OR "Transfer Learning" OR "Learning, Transfer" OR "Language Processing, Natural" OR "Language Processings, Natural" OR "Natural Language Processings" OR "Processing, Natural Language" OR "Processings, Natural Language" OR "Machine Learning") AND ("Adherence, Medication" OR "Drug Adherence" OR "Adherence, Drug" OR "Medication Persistence" OR "Persistence, Medication" OR "Medication Nonadherence" OR "Nonadherence, Medication" OR "Medication Non-Adherence" OR

"Medication Non Adherence" OR "Non-Adherence, Medication" OR "Medication Noncompliance" OR "Noncompliance, Medication" OR "Medication Compliance" OR "Compliance, Medication" OR "Drug Compliance" OR "Compliance, Drug" OR "Medication Non-Compliance" OR "Medication Non Compliance" OR "Non-Compliance, Medication" OR "Administration, Self" OR "Administrations, Self" OR "Self Administrations" OR "Therapeutic Adherence and Compliance" OR "Treatment Adherence" OR "Adherence, Treatment" OR "Therapeutic Adherence" OR "Adherence, Therapeutic" OR "Medication Adherence" OR "adherence monitoring" OR "adherence prediction") AND (Prescription OR Prescriptions OR "Electronic Prescribing" OR "Prescribing, Electronic")

Web of Science (26 articles)

(Patient OR Clients OR "patient participation" OR "Clinic Visits" OR "Clinic Visit") AND ("automated reasoning" OR "Artificial Intelligence" OR "Computational Intelligence" OR "machine learning" OR "Transfer of Learning" OR "Processing, Natural Language" OR "Machine Learning") AND ("Adherence, Medication" OR "medication compliance" OR "Medication Persistence" OR "Medication Nonadherence" OR "Compliance, Medication" OR "Drug Compliance" OR "Administration, Self" OR "Self Administrations" OR "Therapeutic Adherence and Compliance" OR "Treatment Adherence" OR "Adherence, Treatment" OR "Therapeutic Adherence" OR "Adherence, Therapeutic" OR "Medication Adherence") AND (Prescription OR Prescriptions OR "Electronic Prescribing" OR "Prescribing, Electronic")

LILACS (19 articles)

((Patient OR Clients OR Client OR "Clinic Visits" OR "Clinic Visit") AND ("Intelligence, Artificial" OR "Computer Reasoning" OR "Reasoning, Computer" OR "AI (Artificial Intelligence)" OR "Machine Intelligence" OR "Intelligence, Machine" OR "Computational Intelligence" OR "Intelligence, Computational" OR "Learning, Machine" OR "Transfer Learning" OR "Learning, Transfer" OR "Language Processing, Natural" OR "Language Processings, Natural" OR "Natural Language Processings" OR "Processing, Natural Language" OR "Processings, Natural Language" OR "Machine Learning") AND ("Adherence, Medication" OR "Drug Adherence" OR "Adherence, Drug" OR "Medication Persistence" OR "Persistence, Medication" OR "Medication Nonadherence" OR "Nonadherence, Medication" OR "Medication Non-Adherence" OR "Medication Non Adherence" OR "Non-Adherence, Medication" OR "Medication Noncompliance" OR "Noncompliance, Medication" OR "Medication Compliance" OR "Compliance, Medication" OR "Drug Compliance" OR "Compliance, Drug" OR "Medication Non-Compliance" OR "Medication Non Compliance" OR "Non-Compliance, Medication" OR "Administration, Self" OR "Administrations, Self" OR "Self Administrations" OR "Therapeutic Adherence and Compliance" OR "Treatment Adherence" OR "Adherence, Treatment" OR "Therapeutic Adherence" OR "Adherence, Therapeutic" OR "Medication Adherence" OR "adherence monitoring" OR "adherence prediction") AND (Prescription OR Prescriptions))

Supplementary Table 1 - Reports screened studies by title and abstract, excluded with reasons

N	Identification	Authors	Year	No direct patient support for adherence	Support only for prescribers or systems	No answer to the research question#
1	Search strategy	Bayley, K.B. et al[1]	2013	X		X
2	Search strategy	Dixon, B.E .et al[2]	2013		X	X
3	Search strategy	Horsky, J. et al[3]	2013	X	X	X
4	Search strategy	Ogallo, W. and Kanter, A. S.[4]	2016	X		X
5	Search strategy	Lilih, S.et al[5]	2017		X	X
6	Search strategy	Bruce Guthrie[6]	2019	X		X
7	Search strategy	Appold, K.[7]	2019	X		X
8	Search strategy	Chaix, B. et al[8]	2019	X		X
9	Search strategy	Diaz Ochoa, J.G. and Weil, F.[9]	2019	X		X
10	Search strategy	Godin, O. et al[10]	2019	X		X
11	Search strategy	Goh, MLI et al[11]	2019			X
12	Search strategy	Hsieh CY[12]	2019	X		X
13	Search strategy	Rafiei, R.[13]	2019			X
14	Search strategy	MacFarlane, A[14]	2020	X		X
15	Search strategy	Cheon, A. et al[15]	2020			X
16	Search strategy	Lin, A. et al[16]	2020	X		X
17	Search strategy	Oakey-Neate, L. et al[17]	2020			X
18	Search strategy	Omboni, S. et al[18]	2020		X	X
19	Search strategy	Payne, N. et al[19]	2020		X	X
20	Search strategy	Rao, I et al[20]	2020		X	
21	Search strategy	Zhou, N. et al[21]	2020			X
22	Search strategy	Bailly, S et al[22]	2021	X		X
23	Search strategy	Bulaj, G. et al[23]	2021	X		X
24	Search strategy	Gauthier, P. and Cardot, J.M.[24]	2021	X		X
25	Search strategy	Hasan, M.M. et al[25]	2021		X	X
26	Search strategy	Donnenberg, N.S. et al[26]	2022	X		X
27	Search strategy	Liu, E.S.et al[27]	2022	X		X
28	Search strategy	Oh, S.H. et al[28]	2022	X	X	
29	Search strategy	Scarton, L. et al[29]	2022	X	X	
30	Search strategy	Singla, R. et al[30]	2022	X	X	
31	Search strategy	Tahseen, D. and Nambudiri, V.E.[31]	2022	X		X
32	Search strategy	Tran, T.T. et al[32]	2022			X
33	Search strategy	Zhu, Y. et al[33]	2022	X		X

34	Search strategy	Al Kuwaiti, A. et al[34]	2023		X	X
35	Search strategy	Biehl, J et al[35]	2023	X		X
36	Search strategy	Chen, J. et al[36]	2023	X		X
37	Search strategy	Crespo, J. et al[37]	2023	X		X
38	Search strategy	Crilly, P.[38]	2023		X	X
39	Search strategy	Garcia-Manau, P. et al[39]	2023	X		X
40	Search strategy	Jeong, J. et al[40]	2023	X	X	
41	Search strategy	Kiemde, F. et al[41]	2023		X	X
42	Search strategy	Machetanz, L et al[42]	2023	X		X
43	Search strategy	Mucherino, S et al[43]	2023	X		X
44	Search strategy	Narindrarangkura, P. et al[44]	2023	X		X
45	Search strategy	Umar, A.K. et al[45]	2023	X		X
46	Search strategy	Zhang, J.X. and Meltzer, D.O.[46]	2023	X		X
47	Search strategy	Zhang, L. et al[47]	2023	X		X
48	Search strategy	Amiesimaka, O.I. et al[48]	2024			X
49	Search strategy	Asano, M et al[49]	2024	X		X
50	Search strategy	Chart-Pascual, JP et al[50]	2024	X		X
51	Search strategy	Hoehns, J.D. et al[51]	2024		X	X
52	Search strategy	Ke, Z. et al[52]	2024			X
53	Search strategy	Khartabil, N. et al[53]	2024		X	
54	Search strategy	Kostev, K. et al[54]	2024			X
55	Search strategy	Martens, P.J. and Mathieu, C.[55]	2024			X
56	Search strategy	Mehta, S.[56]	2024			X
57	Search strategy	Rammal, D.S. et al[57]	2024	X		X
58	Search strategy	Schliess, F. et al[58]	2024	X		X
59	Search strategy	Zhang, H. et al[59]	2024		X	

*No AI-based tools for medication adherence or mitigating self-administration errors

1. Bayley, K. B. *et al.* Challenges in Using Electronic Health Record Data for CER: Experience of 4 Learning Organizations and Solutions Applied. *Med. Care* **51**, S80–S86 (2013).
2. Dixon Brian E., Jabour Abdulrahman M., Phillips Erin O'Kelly, & Marrero David. G. Improving Medication Adherence for Chronic Disease Using Integrated e-Technologies. in *Studies in Health Technology and Informatics* (IOS Press, 2013). doi:10.3233/978-1-61499-289-9-929.
3. Horsky, J., Phansalkar, S., Desai, A., Bell, D. & Middleton, B. Design of decision support interventions for medication prescribing. *Int. J. Med. Inf.* **82**, 492–503 (2013).
4. Ogallo, W. & Kanter, A. S. Using Natural Language Processing and Network Analysis to Develop a Conceptual Framework for Medication Therapy Management Research. *AMIA Annu. Symp. Proc. AMIA Symp.* **2016**, 984–993 (2016).
5. Lilih, S., Pereboom, M., Van Der Hoeven, R. T. M., Mantel-Teeuwisse, A. K. & Becker, M. L. Improving the effectiveness of drug safety alerts to increase adherence to the guideline for gastrointestinal prophylaxis. *Int. J. Med. Inf.* **97**, 139–144 (2017).
6. Research on multimorbidity in primary care. Selected abstracts from the EGPRN meeting in Tampere, Finland, 9–12 May 2019: All abstracts of the conference can be found at the EGPRN website: www.egprn.org/page/conference-abstracts. *Eur. J. Gen. Pract.* **25**, 164–175 (2019).
7. Appold, K. Pharmacists Embrace New Technologies: The technologies making an impact on pharmacy. *Drug Topics* vol. 163 11+ (2019).

8. Chaix, B. *et al.* When Chatbots Meet Patients: One-Year Prospective Study of Conversations Between Patients With Breast Cancer and a Chatbot. *JMIR Cancer* **5**, e12856 (2019).
9. Diaz Ochoa, J. G. & Weil, F. From personalization to patient centered systems toxicology and pharmacology. *Comput. Toxicol.* **11**, 14–22 (2019).
10. Godin, O. *et al.* Validation and refinement of the clinical staging model in a French cohort of outpatient with schizophrenia (FACE-SZ). *Prog. Neuropsychopharmacol. Biol. Psychiatry* **92**, 226–234 (2019).
11. Goh, M. L. I. *et al.* A Pocket-Sized Interactive Pillbox Device: Design and Development of a Microcontroller-Based System for Medicine Intake Adherence. in *2019 International Conference on Computational Intelligence and Knowledge Economy (ICCIKE)* 718–723 (IEEE, Dubai, United Arab Emirates, 2019). doi:10.1109/ICCIKE47802.2019.9004276.
12. Hsieh, C.-Y. Medication Adherence and Stroke Prevention: What Real World Data Tells Us. *Acta Neurol. Taiwanica* **28(4)**, 86–87 (2019).
13. Rafieri, Ramin. ONdrugDelivery Magazine,. *Macro Trends Accelerating the Adoption of Connectivity in Drug Delivery* vol. 98 24–28 (2019).
14. General Practice and the Community: Research on health service, quality improvements and training. Selected abstracts from the EGPRN Meeting in Vigo, Spain, 17–20 October 2019: All abstracts of the conference can be found at the EGPRN website https://www.egprn.org/page/conference-abstracts. *Eur. J. Gen. Pract.* **26**, 42–50 (2020).
15. Cheon, A. *et al.* A Machine Learning Approach to Detecting Low Medication State with Wearable Technologies. in *2020 42nd Annual International Conference of the IEEE Engineering in Medicine & Biology Society (EMBC)* 4252–4255 (IEEE, Montreal, QC, Canada, 2020). doi:10.1109/EMBC44109.2020.9176310.
16. Lin, A., Stolfi, A., Eicher, T. & Neeley, S. Predicting second-generation antidepressant effectiveness in treating sadness using demographic and clinical information: A machine learning approach. *J. Affect. Disord.* **272**, 295–304 (2020).
17. Lin, A., Stolfi, A., Eicher, T. & Neeley, S. Predicting second-generation antidepressant effectiveness in treating sadness using demographic and clinical information: A machine learning approach. *J. Affect. Disord.* **272**, 295–304 (2020).
18. Omboni, S., Panzeri, E. & Campolo, L. E-Health in Hypertension Management: an Insight into the Current and Future Role of Blood Pressure Telemonitoring. *Curr. Hypertens. Rep.* **22**, 42 (2020).
19. Payne, N. *et al.* Medication Adherence and Liquid Level Tracking System for Healthcare Provider Feedback. *Sensors* **20**, 2435 (2020).
20. Rao, I. *et al.* Predicting and improving patient-level antibiotic adherence. *Health Care Manag. Sci.* **23**, 507–519 (2020).
21. Zhou, N. *et al.* Advanced Data Analytics for Clinical Research Part II: Application to Cardiothoracic Surgery. *Innov. Technol. Tech. Cardiothorac. Vasc. Surg.* **15**, 155–162 (2020).
22. Bailly, S. *et al.* Clusters of sleep apnoea phenotypes: A large pan-European study from the European Sleep Apnoea Database (ESADA). *Respirology* **26**, 378–387 (2021).
23. Bulaj, G., Clark, J., Ebrahimi, M. & Bald, E. From Precision Metapharmacology to Patient Empowerment: Delivery of Self-Care Practices for Epilepsy, Pain, Depression and Cancer Using Digital Health Technologies. *Front. Pharmacol.* **12**, 612602 (2021).
24. Gauthier, P. HEALTH CARE DIGITALIZATION, THE STRAIGHTEST PATHWAY TO PERSONALIZATION. *FARMACIA* **69**, 238–245 (2021).
25. Hasan, M. M. *et al.* A machine learning based two-stage clinical decision support system for predicting patients' discontinuation from opioid use disorder treatment: retrospective observational study. *BMC Med. Inform. Decis. Mak.* **21**, 331 (2021).
26. Donnenberg, N. S., Hernandez, I. & Normolle, D. P. Determining the prevalence and risk factors for prescription drug unaffordability. *Res. Soc. Adm. Pharm.* **18**, 2904–2908 (2022).
27. Liu, E.-S. *et al.* Quality care in ST-segment elevation myocardial infarction. *J. Chin. Med. Assoc.* **85**,

268–275 (2022).
28. Oh, S. H., Lee, S. J. & Park, J. Precision Medicine for Hypertension Patients with Type 2 Diabetes via Reinforcement Learning. *J. Pers. Med.* **12**, 87 (2022).
29. Scarton, L. *et al.* Medication Adherence and Cardiometabolic Control Indicators Among American Indian Adults Receiving Tribal Health Services: Protocol for a Longitudinal Electronic Health Records Study. *JMIR Res. Protoc.* **11**, e39193 (2022).
30. Singla, R., Aggarwal, S., Bindra, J., Garg, A. & Singla, A. Developing Clinical Decision Support System using Machine Learning Methods for Type 2 Diabetes Drug Management. *Indian J. Endocrinol. Metab.* **26**, 44–49 (2022).
31. Tahseen, D. & Nambudiri, V. E. Prescription digital therapeutics in dermatology. *J. Am. Acad. Dermatol.* **86**, 193–194 (2022).
32. Tran, T. T., Richardson, A. J. W., Chen, V. M. & Lin, K. Y. Fast and Accurate Ophthalmic Medication Bottle Identification Using Deep Learning on a Smartphone Device. *Ophthalmol. Glaucoma* **5**, 188–194 (2022).
33. Zhu, Y., Liu, Y. & Jiang, H. Geriatric Health Care During the COVID-19 Pandemic: Managing the Health Crisis. *Clin. Interv. Aging* **Volume 17**, 1365–1378 (2022).
34. Al Kuwaiti, A. *et al.* A Review of the Role of Artificial Intelligence in Healthcare. *J. Pers. Med.* **13**, 951 (2023).
35. Biehl, J. T., Patel, R. & Lee, A. J. Toward the Design of Sensing-Based Medication Adherence Aids That Support Individualized Activities of Daily Living: Survey and Interviews With Patients and Providers. *JMIR Hum. Factors* **10**, e40173 (2023).
36. Chen, J. *et al.* Trajectory of glycated haemoglobin over time, using real-world data, in type 2 diabetes patients with obesity on a U-100 basal-bolus insulin regimen. *Diabetes Obes. Metab.* **25**, 1677–1687 (2023).
37. Crespo, J. *et al.* Current State of Telemedicine in the Field of Hepatology in Spain: Challenges, Threats and Next Steps to Follow for an Intelligent Digital Transformation. *Rev. Esp. Enfermedades Dig.* (2022) doi:10.17235/reed.2022.8918/2022.
38. Crilly, P. Opportunities and threats for community pharmacy in the era of enhanced technology and artificial intelligence. *Int. J. Pharm. Pract.* **31**, 447–448 (2023).
39. Garcia-Manau, P. *et al.* Clinical effectiveness of routine first-trimester combined screening for pre-eclampsia in Spain with the addition of placental growth factor. *Acta Obstet. Gynecol. Scand.* **102**, 1711–1718 (2023).
40. Jeong, J., Han, H., Ro, D. H., Han, H.-S. & Won, S. Development of Prediction Model Using Machine-Learning Algorithms for Nonsteroidal Anti-inflammatory Drug-Induced Gastric Ulcer in Osteoarthritis Patients: Retrospective Cohort Study of a Nationwide South Korean Cohort. *Clin. Orthop. Surg.* **15**, 678 (2023).
41. Kiemde, F. *et al.* A Randomized Trial to Assess the Impact of a Package of Diagnostic Tools and Diagnostic Algorithm on Antibiotic Prescriptions for the Management of Febrile Illnesses Among Children and Adolescents in Primary Health Facilities in Burkina Faso. *Clin. Infect. Dis.* **77**, S134–S144 (2023).
42. Machetanz, L., Hofmann, A. B., Möhrke, J. & Kirchebner, J. Offenders and non-offenders with schizophrenia spectrum disorders: the crime-preventive potential of sufficient embedment in the mental healthcare and support system. *Front. Psychiatry* **14**, 1231851 (2023).
43. Mucherino, S. *et al.* Longitudinal Trajectory Modeling to Assess Adherence to Sacubitril/Valsartan among Patients with Heart Failure. *Pharmaceutics* **15**, 2568 (2023).
44. Narindrarangkura, P., Alafaireet, P. E., Khan, U. & Kim, M. S. Predicting suicide attempts among people with diabetes using a large multicenter electronic health records dataset. *Int. J. Psychiatry Med.* **58**, 302–324 (2023).
45. Umar, Abd. K. *et al.* Telepharmacy: a modern solution for expanding access to pharmacy services. in *Artificial Intelligence, Big Data, Blockchain and 5G for the Digital Transformation of the Healthcare*

Industry 111–150 (Elsevier, 2024). doi:10.1016/B978-0-443-21598-8.00009-9.
46. Zhang, J. X. & Meltzer, D. O. Developing an Integrated Longitudinal Dataset for Patient-Centered Outcome Measures in Cost-Related Medication Nonadherence. *Med. Care* **61**, S139–S146 (2023).
47. Zhang, L. *et al.* Anti-atherosclerotic effects of naringenin and quercetin from Folium Artemisiae argyi by attenuating Interleukin-1 beta (IL-1β)/ matrix metalloproteinase 9 (MMP9): network pharmacology-based analysis and validation. *BMC Complement. Med. Ther.* **23**, 378 (2023).
48. Amiesimaka, O., Aluzaite, K., Braund, R. & Schultz, M. Improving Medication Adherence Levels in Inflammatory Bowel Disease (IBD) Patients: A Narrative Evidence-Based Review. *Patient Prefer. Adherence* **Volume 18**, 905–916 (2024).
49. Asano, M. *et al.* Factor Analysis of Patients Who Find Tablets or Capsules Difficult to Swallow Due to Their Large Size: Using the Personal Health Record Infrastructure of Electronic Medication Notebooks. *J. Med. Internet Res.* **26**, e54645 (2024).
50. Chart-Pascual, J. P. *et al.* Areas of interest and sentiment analysis towards second generation antipsychotics, lithium and mood stabilizing anticonvulsants: Unsupervised analysis using Twitter. *J. Affect. Disord.* **351**, 649–660 (2024).
51. Hoehns, J. D. *et al.* Community Pharmacist and Family Medicine Collaboration for Pre-Visit Planning for Shared Patients Receiving Chronic Care Management Services. *J. Pharm. Pract.* **37**, 571–577 (2024).
52. Ke, Z. *et al.* Acupuncture Point Selection Patterns for Chemotherapy-Induced Nausea and Vomiting: A Data Mining Analysis. *Complement. Med. Res.* **31**, 343–358 (2024).
53. Khartabil, N., Morello, C. M. & Macedo, E. Predictive Modeling of Factors Influencing Adherence to SGLT-2 Inhibitors in Ambulatory Care: Insights from Prescription Claims Data Analysis. *Pharmacy* **12**, 72 (2024).
54. Kostev, K. *et al.* German longitudinal prescription database (LRx): Description of characteristics, use in pharmacoepidemiological research, and limitations. *Int J. Clin. Pharmacol. Ther.* **62**, 20–28 (2024).
55. Martens, P.-J. & Mathieu, C. Type 1 diabetes mellitus: a brave new world. *Nat. Rev. Endocrinol.* **20**, 71–72 (2024).
56. Mehta, S. Exploring angina: A fascinating chat with ChatGPT. *Curr. Probl. Cardiol.* **49**, 102393 (2024).
57. Rammal, D. S., Alomar, M. & Palaian, S. AI-Driven pharmacy practice: Unleashing the revolutionary potential in medication management, pharmacy workflow, and patient care. *Pharm. Pract.* **22**, 1–11 (2024).
58. Schliess, F. *et al.* The German Fast Track Toward Reimbursement of Digital Health Applications: Opportunities and Challenges for Manufacturers, Healthcare Providers, and People With Diabetes. *J. Diabetes Sci. Technol.* **18**, 470–476 (2024).
59. Zhang, H. *et al.* Design and rationale of the Comprehensive intelligent Hypertension managEment SyStem (CHESS) evaluation study: A cluster randomized controlled trial for hypertension management in primary care. *Am. Heart J.* **273**, 90–101 (2024).

Supplementary Table 2 - Reports accessible for eligibility and excluded studies with reasons after full reading

N	Identification	Authors	Year	No direct patient support	Support only for prescribers or systems	Editorials and reviews#
1	Manual	Lee, S..K. et al[1]	2013	X		
2	Search strategy	Lo-Ciganic, W. H. et al[2]	2015	X		
3	Search strategy	Nousias, S. et al[3]	2016	X		
4	Search strategy	Waljee, K. et al[4]	2017	X		
5	Search strategy	Brown, K. J. and Gaggin, H. K.[5]	2019			X
6	Search strategy	Haas, K. et al[6]	2019	X		
7	Search strategy	Galozy, A. and Nowaczyk, S.[7]	2020	X		
8	Search strategy	Aziz, F. et al[8]	2020	X		
9	Manual	Wang, L. et al[9]	2020	X		
10	Search strategy	Gosselin, L et al[10]	2021			X
11	Search strategy	Curto, M. et al[11]	2021			X
12	Manual	Gu, Yiing at al	2021	X		
13	Search strategy	Warren, D. et al[12]	2022	X		
14	Search strategy	Ru, X. Y. et al[13]	2022		X	
15	Search strategy	Alarifi, M. et al[14]	2022	X		
16	Search strategy	Salgado Garcia, F. I. et al[15]	2022	X		
17	Search strategy	Sarraju, A. et al[16]	2023	X		
18	Search strategy	Al Faysal, J. et al[17]	2023	X		
19	Search strategy	Earla, J. R. et al[18]	2023	X		
20	Search strategy	Iyo, M. et al[19]	2023	X		
21	Search strategy	Bakken, S.[20]	2023			X
22	Search strategy	Liu, P.-S. et al[21]	2024		X	
23	Search strategy	Adhikari, S. et al[22]	2023	X		
24	Search strategy	Kanyongo, W. and Ezugwu, A. E.[23]	2023			X
25	Search strategy	Chen, Y.-L. et al[24]	2024	X		

Reviews used only for citation searching

1. Lee, S. K., Kang, B.-Y., Kim, H.-G. & Son, Y.-J. Predictors of Medication Adherence in Elderly Patients with Chronic Diseases Using Support Vector Machine Models. *Healthc. Inform. Res.* **19**, 33 (2013).
2. Lo-Ciganic, W.-H. *et al.* Using Machine Learning to Examine Medication Adherence Thresholds and Risk of Hospitalization. *Med. Care* **53**, 720–728 (2015).
3. Nousias, S. *et al.* Monitoring asthma medication adherence through content based audio classification. in *2016 IEEE Symposium Series on Computational Intelligence (SSCI)* 1–5 (IEEE, Athens, Greece, 2016). doi:10.1109/SSCI.2016.7849898.

4. Waljee, A. K. *et al.* Machine Learning Algorithms for Objective Remission and Clinical Outcomes with Thiopurines. *J. Crohns Colitis* **11**, 801–810 (2017).
5. Brown, K. J. & Gaggin, H. K. 'Drugs Do Not Work on Patients Who Do Not Take Them' Can We Do Better in Patient Adherence? *J. Card. Fail.* **25**, 352–354 (2019).
6. Haas, K., Ben Miled, Z. & Mahoui, M. Medication Adherence Prediction Through Online Social Forums: A Case Study of Fibromyalgia. *JMIR Med. Inform.* **7**, e12561 (2019).
7. Galozy, A. & Nowaczyk, S. Prediction and pattern analysis of medication refill adherence through electronic health records and dispensation data. *J. Biomed. Inform.* **112**, 100075 (2020).
8. Aziz, F. *et al.* Determining hypertensive patients' beliefs towards medication and associations with medication adherence using machine learning methods. *PeerJ* **8**, e8286 (2020).
9. Wang, L. *et al.* Applying Machine Learning Models to Predict Medication Nonadherence in Crohn's Disease Maintenance Therapy. *Patient Prefer. Adherence* **Volume 14**, 917–926 (2020).
10. Gosselin, L., Thibault, M., Lebel, D. & Bussières, J.-F. Utilisation de l'intelligence artificielle en pharmacie : une revue narrative. *Can. J. Hosp. Pharm.* **74**, 135–143 (2021).
11. Curto, M. *et al.* Improving adherence to pharmacological treatment for schizophrenia: a systematic assessment. *Expert Opin. Pharmacother.* **22**, 1143–1155 (2021).
12. Gu, Y. et al. Predicting medication adherence using ensemble learning and deep learning models with large scale healthcare data. Sci. Rep. **11**, 18961 (2021).
13. Warren, D. *et al.* Using machine learning to study the effect of medication adherence in Opioid Use Disorder. *PLOS ONE* **17**, e0278988 (2022).
14. Ru, X., Zhu, L., Ma, Y., Wang, T. & Pan, Z. Effect of an artificial intelligence-assisted tool on non-valvular atrial fibrillation anticoagulation management in primary care: protocol for a cluster randomized controlled trial. *Trials* **23**, 316 (2022).
15. Alarifi, M., Jabour, A., Foy, D. M. & Zolnoori, M. Identifying the underlying factors associated with antidepressant drug discontinuation: content analysis of patients' drug reviews. *Inform. Health Soc. Care* **47**, 414–423 (2022).
16. Salgado García, F. I. *et al.* Using wearable technology to detect prescription opioid self-administration. *Pain* **163**, e357–e367 (2022).
17. Sarraju, A. *et al.* Identifying Reasons for Statin Nonuse in Patients With Diabetes Using Deep Learning of Electronic Health Records. *J. Am. Heart Assoc.* **12**, e028120 (2023).
18. Al Faysal, J. *et al.* An explainable machine learning framework for predicting the risk of buprenorphine treatment discontinuation for opioid use disorder among commercially insured individuals. *Comput. Biol. Med.* **177**, 108493 (2024).
19. Earla, J. R., Li, J., Hutton, G. J., Johnson, M. L. & Aparasu, R. R. Comparative adherence trajectories of oral disease-modifying agents in multiple sclerosis. *Pharmacother. J. Hum. Pharmacol. Drug Ther.* **43**, 473–484 (2023).
20. Iyo, M., Akiyoshi, H., Sekine, D., Shibasaki, Y. & Mamiya, N. An exploratory database study of factors influencing the continuation of brexpiprazole treatment (prescription) in patients with schizophrenia using information from psychiatric electronic medical records processed with natural language processing. *Schizophr. Res.* **255**, 122–131 (2023).
21. Bakken, S. Innovative informatics interventions to improve health and health care. *J. Am. Med. Inform. Assoc.* **30**, 409–410 (2023).
22. Liu, P.-S. *et al.* Optimizing methadone dose adjustment in patients with opioid use disorder. *Front. Psychiatry* **14**, 1258029 (2024).
23. Adhikari, S. *et al.* Cohort profile: a large EHR-based cohort with linked pharmacy refill and neighbourhood social determinants of health data to assess heart failure medication adherence. *BMJ Open* **13**, e076812 (2023).
24. Kanyongo, W. & Ezugwu, A. E. Machine learning approaches to medication adherence amongst NCD patients: A systematic literature review. *Inform. Med. Unlocked* **38**, 101210 (2023).
25. Chen, Y.-L. et al. Machine learning-based prediction of medication refill adherence among first-time

insulin users with type 2 diabetes. Diabetes Res. Clin. Pract. **207**, 111033 (2024).

Supplementary Table 3. Details of AI-based tools

	Solutions	Facial recognition	Alerts	IoT or Wi-Fi	Real-time data tracking	Live calls	Messaging & Notifications	Video recording of patients	Voice-based interactions
Bain EE, et al. 2017 (23)	The AI mobile app (AiCure)	YES	YES	YES	YES	NO	YES	YES	NO
Gracey, B et al., 2018 (25)	Call center / AllazoEngine (proprietary)	NO	NO	YES	NO	YES	NO	NO	NO
Da Silva, et al., 2019(27)(*)	Intelligente integrated system with IoT and smart sensors.	NO	YES	YES	YES	NO	YES	YES	NO
Koesmahargyo et al., 2020(26)	The AI mobile app (AiCure)	NO	NO	YES	YES	NO	NO	YES	NO
Julius, M.S et al., 2021 (29)(*)	ML framework to personalized interventions	NO	NO	?	YES	NO	NO	?	NO
Nayak, A. et AL., 2023 (24)	Voice-based conversational AI application	NO	NO	YES	YES	NO	YES	NO	YES
Aparna, R. et al., 2023(28)(*)	IoT-based system, wireless networks, and ML	NO	YES	YES	YES	NO	YES	YES	NO

(*)Not implemented. (?) Undefined. ML: Machine learning.

23. Bain EE, Shafner L, Walling DP, Othman AA, Chuang-Stein C, Hinkle J, et al. Use of a Novel Artificial Intelligence Platform on Mobile Devices to Assess Dosing Compliance in a Phase 2 Clinical Trial in Subjects With Schizophrenia. JMIR MHealth UHealth. 21 de fevereiro de 2017;5(2):e18.
24. Nayak A, Vakili S, Nayak K, Nikolov M, Chiu M, Sosseinheimer P, et al. Use of Voice-Based Conversational Artificial Intelligence for Basal Insulin Prescription Management Among Patients With Type 2 Diabetes: A Randomized Clinical Trial. JAMA Netw Open. 1º de dezembro de 2023;6(12):e2340232.
25. Gracey, B., Jones, C. A., Cho, D., Conner, S., & Greene, E. Improving medication adherence by better targeting interventions using artificial intelligence-a randomized control study. Value Health. 2018;21:S76.
26. Koesmahargyo V, Abbas A, Zhang L, Guan L, Feng S, Yadav V, et al. Accuracy of machine learning-based prediction of medication adherence in clinical research. Psychiatry Res. dezembro de 2020;294:113558.
27. João Da Silva V, Da Silva Souza V, Guimarães Da Cruz R, Mesquita Vidal Martínez De Lucena J, Jazdi N, Ferreira De Lucena Junior V. Commercial Devices-Based System Designed to Improve the Treatment Adherence of Hypertensive Patients. Sensors. 18 de outubro de 2019;19(20):4539.
28. Aparna R, Aravinda Kashyap KS, K R S, R B S, Rao JE, M S. MediSync: An IoT and ML-Powered Medication Adherence Solution. Em: 2023 7th International Conference on Computation System and Information Technology for Sustainable Solutions (CSITSS) [Internet]. Bangalore, India: IEEE; 2023 [citado 18 de outubro de 2024]. p. 1–6. Disponível em: https://ieeexplore.ieee.org/document/10334070/
29. Sunday Julius M, Rita Alo U, Uchenna Onu F, Ihuoma Akobundu C. Machine Learning Framework to Predict Patient Non-Adherence to Medication using Non-Clinical Data: A Prognosis Approach. Em: Proceedings of the 9th International Conference on Computer and Communications Management [Internet]. Singapore Singapore: ACM; 2021 [citado 18 de outubro de 2024]. p. 98–103. Disponível em: https://dl.acm.org/doi/10.1145/3479162.3479177

Supplementary Information - Extracted data

Title
Authors
Year
Authors
Abstract
Status/Selection
Status/Extraction
Reading Priority
Score
Journal
Keywords
Type
comment
URL
Volume
Pages
DOI
ISSN/ISBN
Importation Date
(I) Patients with prescribed medication by an authorized professional
(I) Prescribed medication for self-administration
(I) Approaches based on Artificial Intelligence tools
(I) Medication adherence or self-administration errors
(E) Artificial Intelligence is used to exclusively avoid prescribers' errors as alert on drug interaction or polypharmacy, to the prescriber’s support on dosage, administration route, and frequency of use.
(E) Inaccessible full publication
(E) Study that does not answer the research question
(E) Digital technology, even using AI, to exclusively remember patients their medication schedule
(E) Editorial, opinião, carta ao editor
(E) System to avoid errors during prescription
(E) System to retrospectively predict medicine adherence without patient support
(E) Others
(E) Reviews and editorials
Country of study (scenario)
Main objective

Study design: Prospective (cohort
Study design: clinical trial
Study design: experimental)
Study design: Cross-sectional
Study design: Retrospective
Study design: database analysis
Study design: Review
Study design: Simulation}
Participants (n)
Scenario : Outpatient
Scenario : Discharge instructions
Scenario : Inpatient
Scenario : Undefined
Scenario : Other
Disease or condition
Age of patients, when specific
Prescribing system : Private system
Prescribing system : Public system
Prescribing system : Undefined
Prescribing system : No-applicable}
System coverage : National
System coverage : Multinational
System coverage : Regional
System coverage : Local (city or specific healthcare units)
System coverage : Undefined
System coverage : Non-applicable}
Artificial Intelligence tools
AI approach (stage of development) : Development only (train and test in the same sample)
AI approach (stage of development) : Development with external validation (validation in other geographic
AI approach (stage of development) : temporal or different database)
AI approach (stage of development) : External validation only
AI approach (stage of development) : Post-market analysis
AI approach (stage of development) : No-applicable}
Model performance of IA approach (accuracy, F1, etc)
Outcome: Medication adherence metrics (quantitative or qualitative indicators)
Outcome: Self-administration errors (quantitative or qualitative indicators)

Supplementary Table 4 - Adherence measurement quality according to Emerge checklist

	Operational definition	Phases of medication adherence	Measurement	Results
Recommendation according to EMERGE(22)	Yes. A precise operational/working definition for each phase of medication adherence studied)	Phase(s) of medication adherence studied: initiation, implementation, and persistence) and justify, where possible, the reasons the study focuses on this/these phase(s).	Yes. Methods of measuring medication adherence (e.g., self-report, claims data, blood sampling, electronic monitoring). Consider each phase studied (i.e., initiation, implementation, and persistence), with details on the performance of the measures (e.g., validity, reliability, and potential bias).	Describe the results of the analysis appropriate to each phase of medication adherence studied (i.e., initiation, implementation, and persistence).
Bain EE, et al. 2017 (23)	Yes. Number of doses captured by the AI platform/number of planned doses.<br><br>Pharmacokinetic sample by drug concentration.	Implementation.	Yes. Computer vision to verify drug ingestion). Plasma drug levels Self-reported adherence via an app or phone call.	AI-based tool ability to detect non-adherence patterns.
Gracey, B et al., 2018 (25)	Yes. Intention-to-treat approach on year-end proportion of days covered (PDC) greater than 80%, calculated by Medicare Star Rating methodology using pharmacy claims.	Implementation.	Yes. Pharmacy claims data. Difference-in-differences (DiD) analysis: Compared adherence changes between intervention and control groups over two consecutive years. AI-targeted intervention effectiveness: whether patients in the AI group had a significantly higher likelihood of achieving adherence compared to traditional or control groups.	Patients in the AI Group were 6.1% more likely to be adherent than those in the Control Group (p=0.04).<br><br>AI-targeted interventions increased adherence likelihood by 7.8% compared to the Traditional Group (p=0.08).
Da Silva, et al., 2019(27)(*)	Yes Sensor-detected medication retrieval and intake actions, and wireless technology inform a gateway of dates, times, and locations of medication intake while monitoring the ingestion by a camera.	Initiation, prototype.	No adherence behaviors were measured in a clinical or home setting.	The AI-based tools successfully identified non-adherence behaviors in a simulated environment.
Koesmahargyo et al., 2020(26)	Yes Proportion medication doses confirmed as ingested using AI-based	Implementation.	Yes. Smartphone app with facial recognition and AI-driven image processing to verify	The ML prediction improves adherence assessment, offering an opportunity for direct

	video monitoring.<br>Daily adherence value = n medications expected / n medications are taken with AiCure.<br>Human review:<br>Daily adjusted adherence value = (n medications expected − n medications with red or orange alerts) / n medications taken with AiCure.		medication intake in real-time.<br>AI-based monitoring.<br>Human review.	support to the patient.
Julius, M.S et al., 2021 (29)(*)	Not declared.	Initiation, framework.	No real medication intake tracking occurred.	The study proposed a novel prognosis-based approach, aiming to provide early intervention strategies for high-risk patients of non-adherence.
Nayak, A. et AL., 2023 (24)	Yes.<br>Mean insulin adherence based on logged data and change in the composite scores of the 3 surveys measuring attitudes toward diabetes, health technology, and medication adherence.	Implementation.	Yes.<br>Self-reported insulin administration using the voice-based conversational AI application.	AI-based tool provided significant improvements in adherence, dose titration frequency, and glycemic control.
Aparna, R. et al., 2023(28)(*)	Yes.<br>Number of pills in the pill bottle and alert the user when the quantity falls below a predefined threshold level, prompting them to refill the bottle.<br>The ioT component will employ pill recognition technology to identify the specific pill inside the bottle.	Initiation, prototype.	Yes.<br>Successful intake of pills as detected by an IoT-enabled smart pillbox, using a weight sensor (200g load cell with HX711) to track changes in pill quantity.	The IoT-based system successfully tracked simulated medication intake.

(*)Not implemented. AI: artificial intelligence. ML: machine learning.

23. Bain EE, Shafner L, Walling DP, Othman AA, Chuang-Stein C, Hinkle J, et al. Use of a Novel Artificial Intelligence Platform on Mobile Devices to Assess Dosing Compliance in a Phase 2 Clinical Trial in Subjects With Schizophrenia. JMIR MHealth UHealth. 21 de fevereiro de 2017;5(2):e18.
24. Nayak A, Vakili S, Nayak K, Nikolov M, Chiu M, Sosseinheimer P, et al. Use of Voice-Based Conversational Artificial Intelligence for Basal Insulin Prescription Management Among Patients With Type 2 Diabetes: A Randomized Clinical Trial. JAMA Netw Open. 1º de dezembro de 2023;6(12):e2340232.
25. Gracey, B., Jones, C. A., Cho, D., Conner, S., & Greene, E. Improving medication adherence by better targeting interventions using artificial intelligence-a randomized control study. Value Health. 2018;21:S76.
26. Koesmahargyo V, Abbas A, Zhang L, Guan L, Feng S, Yadav V, et al. Accuracy of machine learning-based prediction of medication adherence in clinical research. Psychiatry Res. dezembro de 2020;294:113558.
27. João Da Silva V, Da Silva Souza V, Guimarães Da Cruz R, Mesquita Vidal Martínez De Lucena J, Jazdi N, Ferreira De Lucena Junior V. Commercial Devices-Based System Designed to Improve the Treatment Adherence of Hypertensive Patients. Sensors. 18 de outubro de 2019;19(20):4539.

28. Aparna R, Aravinda Kashyap KS, K R S, R B S, Rao JE, M S. MediSync: An IoT and ML-Powered Medication Adherence Solution. Em: 2023 7th International Conference on Computation System and Information Technology for Sustainable Solutions (CSITSS) [Internet]. Bangalore, India: IEEE; 2023 [citado 18 de outubro de 2024]. p. 1–6. Disponível em: https://ieeexplore.ieee.org/document/10334070/
29. Sunday Julius M, Rita Alo U, Uchenna Onu F, Ihuoma Akobundu C. Machine Learning Framework to Predict Patient Non-Adherence to Medication using Non-Clinical Data: A Prognosis Approach. Em: Proceedings of the 9th International Conference on Computer and Communications Management [Internet]. Singapore Singapore: ACM; 2021 [citado 18 de outubro de 2024]. p. 98–103. Disponível em: https://dl.acm.org/doi/10.1145/3479162.3479177
